# Opera Minora

**Published:** 1838

**Authors:** 


### Art. VIII.—Opera Minora.

Under this head we propose to notice a number of Discourses, Lectures and short Treatises which have accumulated on our table.

1. *Introductory Address to the Students of Medicine of the College of Physicians and Surgeons of the University of the State of New York.* Delivered Nov. 7, 1837, By Edward Delafield, M. D. Professor of Obsteterics &c.

The respectable author of this Address is more extensively known by his edition of Mr. Travers' on the Eye, than by his teachings in the University. He is evidently a man of sound sense, and writes in a plain and perspicuous style. We are happy to find him insisting on the necessity of a better preparatory education than our students generally receive. We might have supposed, that the students of the Empire State, *do* come up to the study of Medicine with larger preliminary acquirements than those of the newer and wilder West; but from the earnestness with which the Professor presses the subject of elementary education, there can be no doubt that it is often exceedingly defective there, as well as here.

Our Author's advice to young men after they have entered on their professional studies, is highly judicious, though not, of course, very new or original. One paragraph appears to us so important, as to merit re-publication. In speaking of the study of the Materia Medica, he remarks—

"And here, too, the student will do well to use every means of gaining knowledge by his own experience. He cannot too soon engage in preparing medicines for administration. He thus becomes familiar with the substances he is afterwards to prescribe; he learns the modes of detecting their adulterations and impurities; and he employs the very best method of ascertaining and fixing in his memory their doses. There is no part of the education of students of medicine more neglected than this; and I believe it is not very uncommon for gentlemen to graduate, who have never prepared a dose of medicine, nor seen, except in the Lecture Room, half of the more common substances employed as remedies. It is, on this account, almost to be regretted, that the old practice, in this city, of physicians dispensing their own medicines, has fallen into disuse. Their pupils lose an advantage which they can in no other way obtain. I have always felt that the time which I spent, when a student, in preparing the medicines for the patients of my instructor, was as usefully employed as any which was devoted to my medical education."

We should be pleased to see many such introductories as this, printed and disseminated every year.

2. *An Essay on the Relation between the Respiratory and Circulatory Functions.* By Charles Hooker, M. D., read at the Annual New-Haven county meeting, of the Connecticut Medical Society, April 12th 1838.

This Essay of 47 pages, on a subject of deep pathological interest, manifests, on the part of its Author, a commendable degree of research, and a full perception of the importance of his subject. The following page sets forth the results of his inquiries, into the natural ratio between the respiration and the pulse.

"This ratio is stated by Haller to be as 1 to 3 or 4; by Dr. Graves, as 1 to 4. The number of respirations in a minute, in a healthy adult, is estimated by Magendie as 15; by Dr. Dunglison, about 18; by Sir Humphry Davy, 26 or 27; while Dr. Good, Dr. C. J. B. Williams, and most other writers, give 20 as the ordinary number. Supposing the latter to be the true number, and the pulsations, as commonly estimated, to be 70 in a minute, the ratio will be 1 to 3 1-2; while, according to the estimate of Sir Humphry Davy, the ration is about 1 to 2 1-2.

This discrepancy of statements plainly shows, that the observations of authors on this point have been very limited. The only method which will lead to correct conclusions, a method which I have frequently practised since my attention was turned to this subject, is to count the resperation of persons who are not aware of such observation: for, as the respiration is much under the control of the will, its frequency will be varied by the operation of the mind. Hence, a conclusion drawn from observing one's own respiration would be liable to error. Perhaps diversity of climate, and national peculiarities of constitution, may occasion some variation from the ratio which I have stated; but so constant has been this ratio, of 1 to 2 1-2, according to my observation, that I have regarded any considerable variation from it as a pretty sure indication of malformation or disease. In a diagnostic and pathological point of view, therefore, I regard the *comparative frequency* of the respiration and the pulse as highly important.

In early infancy there is less regularity in this ratio. Owing to imperfect development of the lungs, or some other cause, it is not uncommon that an infant, with a pulse of 120 or 130, will have 40, 50, or even 60 respirations in a minute. Generally, however, the healthy ratio becomes established in the course of the first or second year. So in adults, the respiration is rendered frequent by many circumstances which can hardly be considered as diseased. Obesity, by preventing a free and large expansion of the chest, gives occasion to increased frequency of the respiration. The same effect is produced by a distension of the stomach or intestines, by pregnancy in females, and by any circumstance which prevents a free descent of the diaphragm. Any circumstance indeed, that prevents a full quantity of air from being received into the lungs with each inspiration, necessarily calls for more frequent respiration. As a general rule, if the respiration is deficient in fullness, the deficiency is compensated for by increased frequency."

Our limits preclude us from a full analysis of Dr. Hooker's essay, but we will give his results as presented into the closing pages.

### Concluding Summary.

"The preceding essay, it is believed, establishes several important pathological principles, affording valuable diagnostic and theraputic indications, which hitherto have been but slightly noticed, or wholly unknown. The indications of the pulse have received much attention; but the variations of the respiration have been little attended to, and the relations between the respiratory and circulating functions have been almost wholly neglected.

The *comparative frequency* of the respiration and the pulse in health, which from constant observation, during a period of several years, I have ascertained to be 1 to 4 1-2, has not been commonly observed; and most of the indications afforded by *variations of this ratio* have been altogether overlooked.

A disproportionate *increased* frequency of the respiration has been shown to afford the general indication that there is some impediment to the respiration; which may be owing to, A. *Disorder of the lungs or air passages*, as pneumonitis, phthisis, œdema of the lungs, or any affection of the lungs which prevents a portion of them from being freely permeated with air, or any disorder of the bronchia or bronchial membrane which impedes the communication between the air and the blood within the lungs: or, B. Some *mechanical impediment* to the motions of respiration: or, C. *Imperfect function of the organic nerves* of the lungs.

A disproportionate *diminished* frequency of the respiration, which indicates *a want of energy in the nerves which control the respiratory motions*, has been shown to be common typhous fever, and in many other diseases.

The pathological effects of imperfect aeration of the blood, which had been treated of by Bichat and some subsequent writers, but which they scarcely noticed except as immediate precursors and causes of death, I have observed to be manifest through the progress of typhous fever, and many other diseases. What is commonly termed *congestion in the brain*, I have endeavored to show, is simply a deterioration of the blood caused by this imperfect aeration, a prominent example of which occurs in the disease termed congestive typhus. The effects of this imperfect aeration, depending upon disordered function of the different nerves concerned in respiration, have been traced in various diseases.

The common occurrence, and the injurious effects, of this imperfect aeration of the blood suggests the important general *therapeutic indication to remedy deficient respiration.* The medicinal agents are detailed which aggravate deficient respiration, by increasing the circulation, or by diminishing the respiratory function.

The use of remedies, with a view *to promote the arterialization of the blood*, it is believed, has never been distinctly treated of by any author, as a prominent object of medication. Though my 1st class these remedies—those which diminish the action of the heart and arteries—have been commonly known to possess this power over the circulation, still they have not been commonly employed with the view—a view which I consider as highly important in many cases—to obviate a disparity between the respiratory and circulating functions. The 2d and 3d classes of remedies—those which excite and invigorate the motor respiratory nerves, and the arterializing nerves of the lungs—have rarely, if ever, been recommended for those particular purposes; tho' I think it will be obvious to my readers, that many of the known valuable effects of these remedies are owing to such operations. The other three classes—4th. Ventilation; 5th. Remedies which obviate mechanical impediments to the respiration; and, 6th. Remedies which excite secretions vicarious of respiration—though their general effects on the respiratory function have been known, have not been commonly employed for the distinct purpose of obviating deficient aeration of the blood.

In short, the general subject of the pathological relations between the respiratory and circulating functions has received little, very little attention. The writer hopes that he has at least shown the subject to be deserving of investigation."

3. *An Address delivered before the Medical Society of Tennessee at the eighth annual meeting at Nashville, on the 7th of May*, 1838, By Lunsford P. Yandell, M. D.

Professor Yandell of the Louisville Medical Institute, one of the seceding members of the Medical Faculty of Transylvania University, is a native of Tennessee, and was therefore, apart from his acknowledged talents, not unaptly selected by the society, to deliver its annual discourse. A leading, we might say *the* leading, idea of this production, is the contaminating association, in all ages, of superstition with medicine. The illustrations are, therefore, historical, and do not present the freshness which the orator might have spread over his address, had he given it a more indigenous character. We are advocates for the West, as fruitful in topics for profitable discussion; and entirely satisfied, that our author, is in all respects, qualified to treat any of them with success. The address, however, is not without allusions to the present and prospective condition of his native state, in the course of which the author makes the following appropriate appeal to his brethren of the Society.

"In the establishment of this improved order of things, it was the reasonable expectation of the founders of this Society, embracing as it does, a large share of the science of the State, that it would act a distinguished part. Its great purpose has never appeared to me, as it seems to have done to its members generally, to be the regulation of the practice of physic in Tennessee. Assuredly this is a most important object. A most desirable state of things will be that, in which the public will demand some test of qualification in medical practitioners. It will be a new era in the history of man, when he ceases to believe that to cure diseases, like Dogberry's reading and writing, comes by nature. And, in the distant future, the philanthropist has a glimpse of this perfection of human discernment. It will arrive with the completion of our high task—it will come when medical men are agreed touching all the great principles of health, disease, and cure—when they cease to impute to each other dishonest motives for their honest differences—when we shall have discovered the true key to the Temple of Health. There will be no contrariety of opinion then; but the multitude will wait assiduously upon our steps, and press eagerly to the doors of the Temple—and the voice of the empiric will be heard in our streets no more. Mean time, independently of the regulation of practice, there is ample room and verge enough for the exercise of all the energies of the Society. Innumerable topics, deeply interesting to medical men—vitally important to the community, invite their investigation. Mortuary records, meteorological registers, the statistics of our most prevalent diseases—these remain to be furnished by the practitioners of the State, and it is the province of this Society to bring them out. Tennessee remains without a retreat for its insane, but it is gratifying to perceive that this cannot be mnch longer said. Humanity, as well as State dignity, calls for the early completion of this charity. The want of it has been the cause of great and manifold suffering. In all parts of the State, unfortunate beings, too poor to gain admittance into the asylum of a neighboring State, are chained in dark rooms, and log cabbins, and treated with kindness or cruelty, as their keepers or friends may chance to be intelligent and humane, or ignorant and unfeeling."

4. *Designs Nos. 1 and 2, for Marine Hospitals on the Western Waters.* By Robert Mills, Architect.

The readers of the Western Journal are aware, that it was the first periodical, either East or West, that stood forth as the advocate of Marine Hospitals in the latter. Several years have elapsed since, at the suggestion of Dr. Campbell of St. Louis, the subject was first broached. As might have been anticipated, the action of Congress has been tardy and ineffectual. *Something*, however has been done, but what, we are not exactly informed. The sites of several, it is said have been selected, and the *designs*, now before us, have been distributed, at Washington, by an architect, apparently for the inspection of the government. They are two in number, one for the accommodation of one hundred patients, the other for half the number. It is not our intention to examine them, but to express our opinion in favor of the smaller. Except in New-Orleans, it can never be necessary to have Commercial Hospitals in the West, of greater size than will accommodate fifty patients. By this limitation, the number of establishments may be much larger at the same cost, than if a more extended plan should be adopted, and the great *desideratum* is their multiplication. We ask the attention of our brethren to this view of the matter.

5. *Fiske-Fund Prize Dissertation of the Rhode-Island Medical Society, on Cholera Infantum, its Causes and Treatment.* By David King, Jr. M. D. June 1837. Boston.

We make from the 6th page of this dissertation, the following extract.

"*Causes of Cholera Infantum.*—In the first place, this disease has a specific miasmatic cause. Most endemic maladies, probably, arise from some emanation from the soil, owing to the dissolution of animal and vegetable matter. We know not the nature of these miasms, because they are beyond the reach of our senses and the analyzing process of art. It is probable, however, that, at first, the animal and vegetable matter is decomposed into atoms of effluvia; and that these atoms of effluvia enter, afterwards, into those peculiar combinations which constitute specific miasms. Our knowledge of the origin of febrile miasm consists, chiefly, in the established fact, that for its production are required a combination of four elements—animal or vegetable matter, atmospherical air, a high temperature, and water in a moderate quantity. But the circumstances of temperature and moisture, elevation, texture and depth of soil, which determine the specific form of the febrile miasm, are beyond the reach of our observation and experiment. We do not know all the combined causes required to produce "hepatitis on the coast of Coromandel, elephantiasis in Malabar, beriberi in Ceylon, Barbadoes leg in the Antilles, goitre among the Alps, the plica in Poland, cretinism in the Vallais," or cholera infantum in the large cities of the United States. The existence of the febrile miasm, producing cholera infantum, is known by its effects. It is confined to particular localities, supplied with materials for the production of miasm. Were the disease attributable to common causes, as heat, moisture, and atmospherical vicissitudes, this pestis infantum would be a pervading disease, through the whole range of the United States. But its great source is to be found, only, in our large cities, where heat, moisture, a semi-stagnant atmosphere, and filth, or animal and vegetable remains, spread over a large surface, readily produce the *malarious emanata.*"

Here we have a most happy generalization of hepatitis, beriberi, sore-leg, goitre, plica polonica, cretanism, and cholera infantum, under one unknown cause, modified by other unknown causes! Miasma, requires we are told, for its production, a combination of four elements—animal or vegetable matter, atmospherical air, a high temperature, and water in a moderate quantity; but where are these elements most abundant, in town or country? Undoubtedly in the latter, while cholera infantum prevails most in the former. Where is ague and fever most prevalent? Certainly in the country, and if *any* disease arises from malaria, it must be that fever. We can by no means grant, that our author has *established* the relation of cause and effect between malaria and cholera. For ourselves, we have seen but one circumstance, which appeared to stand in the relation of cause to that malady, and it is excessive heat. In Cincinnati, we have always observed it to begin after the few first days of intense heat. It frequently *prevails* in the latter part of May; and endures through June and a part of July. *New* cases rarely occur after the middle of that month, and some times, not after the end of June. But why should not the heat of the surrounding country induce the disease? All we can say on this point is, that it does, when it is intense; but in the same latitudes, the heat of cities is generally greater than that of the country. We have seen very violent, and even fatal cases, in the country, where the soil and aspects were in no degree favorable to the development of miasma; but where and when the heat was intense.

"The peculiar miasm, which produces cholera infantum, acts upon the minute ramifications of the ganglionic nerves, in the lungs, and by means of the blood through the vascular and capillary systems. This primary influence of the miasm on the organic nerves is succeeded by excessive secretory irritation of the follicles of the mucous membrane of the alimentary canal, which constitutes the disease."

We should like to see a statement of the facts from which these conclusions are drawn.

About one third of Dr. King's dissertation is devoted to the treatment of Cholera Infantum. We had hoped, in this part, to have met with something new, but are disappointed. The whole presents little else than a loosely arranged collection of the remedies employed by Kuhn, Rush, Dewees, Parish, Chapman, Caldwell, Eberle, and Cartright, with so few of the authors own observations and experiments, as to disclose, that he has had but little practice in the disease, on which he has undertaken to write a prize dissertation. We cannot even speak with full approbation of the means he has adopted. They are too numerous, complicated, and bulky. Why did not our author quote the admirable little paper of Dr. Edward Miller of New-York, published nearly *thirty* years ago. Judging from our own experience, the small doses of calomel and opium recommended in that paper, are worth every thing else, and ought to supersede almost all other means, in the treatment of this malady. When fever is present, the opium should be omitted and calomel only administered. When the discharges are profuse, and exhaustion is threatened, the quantity of opium thrown in, should be such as to produce early and protracted, narcotism. This compound, with a blister or sinapism to the epigastrium, cold applications to the head and abdomen, and great restriction in drinks, make up nearly the whole of the treatment which we have at any time found beneficial.

It is far from our desire to speak disparagingly of Dr. King's dissertation. It is undoubtedly a respectable compilation, and would have made a creditable inaugural thesis. We cannot, however, comprehend the propriety of awarding prizes for such essays. Prize-money should be made to meet the expense of original experiments; not of books, to furnish excerpts. We trust that Dr. King will unite with us in this sentiment, and that he will direct his efforts upon the production of new facts—it is they only that enlarge the boundaries of our science.

6. *Statistical Observations on the number of blind in Pennsylvania and the United States.* By SAMUEL HAZARD, Esq., of Philadelphia.

[**Transactions of the Medical Society of the State of New-York. 1838.**]

Our friend Hazard has condensed into those few pages a great amount of statistical information, on a deeply interesting subject. It is quite impossible to abridge his paper, and our limits do not admit of its republication, but we shall transfer to our Journal a few of its first pages entire.

The tabular view he has given ,presents a melancholy aggregate of blind persons, and still, we are convinced, it falls short of the reality. What is the duty of the profession and of society on this subject? It is to make more ample preparation for the treatment of diseases of the eye, and for the education of those who are permanently blind. It must be admitted that a great many physicians, who, as general practitioners are respectable, fall short in the knowledge of the anatomy, physiology and diseases of the organ of vision. We affirm the fact without inquiring into the cause; and we pronounce it discreditable, without stopping to consider on the *policy* of our denunciation. At the same time, it must be confessed, that such an amount of special preparation is necessary to the successful treatment of ophthalmic diseases, that without the spirited co-operation of society, the profession cannot accomplish all which should be done. Endowed Infirmaries should be instituted in all our principal towns, where those who labor under chronic maladies of the eye, may be assembled, and placed in the circumstances, without which they are seldom cured. It is almost as necessary to take away the liberty of this class of patients, as if they were lunatics. Their appetites are generally good, their locomotive powers unimpaired and their visual instincts so strong, that they will, unless physically restrained, perpetually violate almost every rule laid down for their government. But we must come to the table which Mr. H. has compiled with so much care and labor.

*Table showing the number of Blind in the United States: also, the relative proportion to the population, &c.*

| STATES. | Total No. Blind. | Proportion to whole population—1 to | Proportion of blind whites to whole white population—1 to | Proportion of colored blind to whole colored population—1 to | Proportions of colored to the whole population. |
|---|---|---|---|---|---|
| Maine, | 160 | 2497 | 2505 | 1177 | 1 in 339 |
| New-Hampshire, | 105 | 2565 | 2559 | ...... | 1 " 443 |
| Massachusetts, | 223 | 2737 | 2768 | 1409 | 1 " 86 |
| Rhode-Island, | 64 | 1518 | 1672 | 447 | 1 " 27 |
| Connecticut, | 195 | 1526 | 1540 | 1152 | 1 " 30 |
| Vermont, | 51 | 5503 | 5485 | ...... | 1 " 317 |
| New-York, | 724 | 2650 | 2918 | 547 | 1 " 43 |
| New-Jersey, | 227 | 1413 | 1464 | 734 | 1 " 16 |
| Pennsylvania, | 503 | 2680 | 2758 | 1369 | 1 " 35 |
| Delaware, | 29 | 2646 | 3205 | 1741 | 1 " 4 |
| Maryland, | 271 | 1649 | 1980 | 1257 | 1 " 3 |
| Virginia, | 793 | 1527 | 1956 | 1180 | 1 " 2 |
| North-Carolina, | 384 | 1922 | 2120 | 1647 | 1 " 3 |
| South-Carolina, | 238 | 2442 | 2528 | 2377 | 1 " 2 |
| Gerogia, | 273 | 1893 | 1979 | 1789 | 1 " 2 |
| Alabama, | 116 | 2568 | 2800 | 2482 | 1 " 2 |
| Mississippi, | 56 | 2439 | 2817 | 2135 | 1 " 2 |
| Louisiana, | 113 | 1909 | 2485 | 1640 | 1 " 2 |
| Tennessee, | 213 | 3281 | 3044 | 3950 | 1 " 4 |
| Kentucky, | 252 | 2729 | 3064 | 2050 | 1 " 3 |
| Ohio, | 238 | 3940 | 3993 | 1596 | 1 " 98 |
| Indiana, | 87 | 3942 | 3887 | 1816 | 1 " 94 |
| Illinois, | 39 | 4037 | 4443 | 596 | 1 " 66 |
| Missouri, | 37 | 3766 | 4251 | 2566 | 1 " 5 |
| Michigan, | 5 | 6327 | 6269 | ...... | 1 " 108 |
| Arkansas, | 10 | 3038 | 3209 | 2358 | 1 " 6 |
| Florida, | 19 | 1828 | 6128 | 1020 | 1 " 2 |
| District of Columbia, | 19 | 2096 | 2506 | 1534 | 1 " 3 |
| | 5444 | 2363 | 2650 | 1584 | |

"Upon a review of the above table, our attention is first directed to the great difference which exists in the relative proportions of the blind to the population, in the several States, varying from 1 in 1413 to 1 in 6327. The greatest proportion of blind persons being in New-Jersey, and the smallest in Michigan.

On comparing the proportion of the *white* blind with the *colored* blind, the difference is still more remarkable; the largest proportion of the former, being in New-Jersey, viz: 1 in 1464, and the smallest in Michigan, 1 in 6269; while the largest proportion of the *colored* is in Rhode-Island, 1 in 447, and the smallest in Tennessee, 1 in 3950.

In the State of Tennessee alone, is there are an excess in the proportion of *white* blind over the *colored*, the former being 1 in 3044, and the latter 1 in 3950. In South-Carolina, the proportion is nearly equal, the *whites* being 1 in 2528, and the *colored* 1 in 2377. In Alabama and Georgia, also, the proportions are not so different as in the other States—the *whites* being in the former 1 in 2800, and the *colored* 1 in 2482, and in the latter 1 in 1979 for the *whites*, and 1 in 1789 for the *colored*. In most of the other States, the excess in the proportion of the *colored* is very remarkable.

In the whole population of the United States, the proportion of blind is 1 in 2363—of the *white* blind to the *white* population 1 in 2650, and of the *colored* blind to the *colored* population 1 in 1584.

How are these differences to be accounted for?

Geographical position may not fully explain it, yet some facts in this respect are worthy of notice.

Those States on the Atlantic coast which are more fully exposed to the influence of the sea air, have the largest proportion of blind; as for example:

| | |
|---|---|
| Rhode-Island, - - - | 1 in 1518 |
| Connecticut, - - - - | 1 in 1526 |
| New-Jersey, - - - | 1 in 1413 |
| Maryland, - - - - | 1 in 1649 |
| Virginia, - - - - | 1 in 1527 |
| North-Carolina, - - - | 1 in 1922 |
| Georgia, - - - - | 1 in 1893 |
| Florida, - - - - | 1 in 1828 |
| Louisiana, - - - | 1 in 1909 |

The proportions diminishing as we proceed South.

As we proceed into the interior from the sea-coast, the proportion decreases still farther, as for instance.

| | |
|---|---|
| Pennsylvania, - - - | 1 in 2680 |
| Kentucky, - - - | 1 in 2729 |
| Tennessee, - - - - | 1 in 3201 |
| Arkansas, - - - | 1 in 3038 |
| Missouri, - - - - | 1 in 3796 |

Proceeding still further from the sea-coast, and approaching those great reservoirs of fresh water, the lakes, a further decrease in the proportion occurs, as for example—

| | |
|---|---|
| Indiana, - - . - | 1 in 3942 |
| Ohio, - - - | 1 in 3940 |
| Vermont, - - - - | 1 in 5503 |

Michigan, (surrounded by lakes) 1 in 6327

In those States, a portion only of whose territory is situated on the Atlantic, while a principal portion of it extends into the interior, or borders on the lakes, the proportion of the blind is about a medium between the two extremes; for instance—

| | |
|---|---|
| Maine, - - - - | 1 in 2497 |
| New-Hampshire, - - - | 1 in 2565 |
| Massachusetts, - - - | 1 in 2737 |
| New-York, - - - - | 1 in 2650 |
| Delaware, . - - | 1 in 2646 |
| South-Carolina, - - - | 1 in 2442 |
| District of Columbia, - - | 1 in 2096 |
| Mississippi, - - - - | 1 in 2439 |
| Alabama, - - - | 1 in 2668 |

It is proabable, therefore, from the foregoing facts, that if a comparison were made, between the portions of these States situated on the sea-coast, and those portions more remote from it, the proportions of the blind, within the influence of the sea-air, would be in excess. This is verified so far as we have made the examination."

7. *A visit to the Red Sulphur Spring of Virginia during the summer of* 1837, *with observations on the waters.* By HENRY HUNTT, M. D.

Three or four years since we noticed a pamphlet by Dr. H. on change of climate in Pulmonary Consumption, and on the use of the waters of the Red Sulphur, on the treatment of that malady. The pamphlet now before us contains many additional observations on their efficacy in diseases of the lungs. They are adapted to states of subacute inflammation whether connected or not with tuberculation of the organ. Passing by many cases illustrative of their power in this respect, we shall extract such paragraphs as present the results of Dr. Huntt's experience and inquiries.

"The Red Sulphur Water is decidedly sedative in its effects. It subdues chronic inflammation, tranquilizes irritation, and reduces the frequency of the pulse in the most astonishing manner.

It has been considered peculiarly adapted to the cure of pulmonary diseases, and it is true, that it has a most beneficial influence in most cases of this disease; but its good effects equally extend to all cases of sub-acute inflammation, whether seated in the stomach, liver, spleen, intestines, kidneys, bladder, and most particularly in the mucous mem brane. In fact, nature never yet gave to man, a remedy, capable of more extensive application, nor better calculated to relieve a larger class of diseases."

"During my visit to the Red Sulphur, every day was devoted to the investigation of the various diseases which afflicted the visiters at that place; noting particularly the effects of the water in different diseases.

Most of the cases, were various forms of pulmonary consumption. In the earliest stage of tuberculous disease the patients generally complained of abdominal plethora with cough, some oppression, and restless nights, with frequent pulse. In all these cases, where the water was taken in such quantities as to operate on the bowels for a week or ten days, and afterwards increasing the quanity so as to act freely as a diuretic, and the patients were abstemious in their diet, and took exercise regularly, a rapid improvement was most generally the consequence. On the contrary, those who used but little exercise, and indulged their appetite without restraint, were slow and tedious in their convalescence. Let it be impressed on the mind of all tuberculous patients, that *sedentary habits* are among the most powerful causes of tuberculous diseases.

Many persons arrive at the Red Sulphur, who are not prepared to use the water, in consequence of high inflammation, or congestion of the lungs, or other organs, attended with pain in the side, constriction at the breast, or hot, and restless nights, with a quick, sharp pulse; all such cases must have the vascular excitement subdued, before the water can be taken with any advantage. I saw several of those cases under the management of *Dr. Saunders*, the resident physician of the place who treated them very successfully, by means of bleeding, local and general—emetics of ipicac before bed-time—blisters, and occasionally the blue pill.

Most of the visiters at the Red Sulphur this season were laboring under tuberculous consumption, of the second, or middle stage, many of them had visited the Spring one or two seasons, and there was scarcely an exception among them, who had not experienced one or more attacks of hœmoptysis; *hœmoptysis* may generally be considered as an indication of tubercles in the lungs. Those who had visited the Spring before, would say, that they returned home apparently cured, cough, night sweats, expectoration, frequent pulse, all relieved —a good appetite restored, and flesh increasing daily. Towards the spring season the pulmonary symptoms would commence to kindle up again, and by June or July, it would become necessary to repeat the visit to the Red Sulphur; although the symptoms were much less aggravated, and the constitution much less enfeebled than during the previous season.

The water of the Red Sulphur seems to act by *soothing* irritation, lessening the frequency of the pulse, and by subduing the inflammation of the tissues in contact with the tubercles; and thereby renderring the tubercles harmless; and also by suspending that tendency of the system to generate, or deposite tuberculous matter.

The cases generally, laboring under this stage of pulmonary disease, improved in their health, particularly, if they remained long enough at the Spring, restricted themselves to a proper diet, and took sufficient exercise; but there were a few among them, who took little, or no exercise, and gave unlimited indulgence to an inordinate appetite. In such cases, I took no interest, and observed but little change in their appearance.

On examining the visiters laboring under pulmonary disease, I observed that all those patients who drank the water so as to act freely on the bowels, for any length of time did not improve in their health, because, active purging is not proper for the lungs in this disease. The water must be drank in such quantities as to act freely on the kidneys.

There seems to be an intimate association between the lungs and the kidneys, and the kidneys seem to be the great emunctories by which the lungs are relieved in all pulmonary diseases; this idea has been repeatedly suggested to me, in my attendance on patients laboring under this disease; on enquiring into their condition, they have frequently said, "I feel much better to-day, I have had a most copious flow of urine which has afforded me great relief." This view of the connexion between the lungs and kidneys, has been confirmed by witnessing the diuretic effects of the Red Sulphur Water in pulmonary diseases—I have a friend who is a physician, and who has labored, more or less, under pulmonary disease for twenty years. He informed me that whenever his lungs were disturbed by irritation, he always resorted to "cooling diuretic medicines for relief."

There were but few persons laboring under the third or last stage of tuberculous disease, who visited the Red Sulphur this season, and among those few, there was scarcely a case that derived any advantage from the use of the water. When tuberculous disease arrives at this stage, and the constitution is broken down, it is not only *useless* but *cruel* to send the patient to the Red Sulphur. I am sorry to say, that several of my patients in this condition, by my advice, visited the Red Sulphur this season, and I witnessed the bad effects of the water in their cases, as well as in the cases of others of a similar character. They were laboring under that peculiar irritation, and perhaps ulceration of the bowels, so common in this stage of the disease; they were unable to drink but a small quantity of the water, and the consequence was, that the bowels were purged and griped, the secretion of the kidneys was not increased, and the patients grew worse daily."

8. *Memorial from the Pennsylvania Lyceum, (to Congress) asking an appropriation to aid in the advancement of Meteorology.* Read and referred to a select committee of April 20th, 1838.

If we have not made known the labours of Prof. Espy, of the Franklin Institute, Philadelphia, on the subject of the cause of storms of rain, hail, and snow, it is not because we underrate their value and interest. We are not informed whether Congress has made an appropriation for the purchase of the instruments referred to in this memorial, and its appended documents. We trust the memorialists will ultimately succeed, and to their aid we would invoke our readers generally. In the following extract from their memorial, the object in view and the means solicited are set forth.

"From all these facts, in connection with those detailed in our previous reports, it is, we think, abundantly proven that the air moves inwards towards the centre of great rains, and consequently upward in the region of the cloud. Nor is this inward motion difficult to account for. It has been long known that the barometer stands low in the middle of great storms, and nothing is more plain than that the air will run on all sides toward the point where the barometer stands lowest, with a velocity proportional to the square root of the depression. And as this air mounts upwards over the point where the barometer stands lowest, a condensation of the vapor must take place, from the cold produced by diminishing pressure as it ascends. Now, the chairman of this committee has shown, by calculation from acknowledged data, that for every pint of water which is formed by condensation of vapor, the cloud itself is expanded 7,600 pints by the latent caloric given out at the moment of condensation. Thus will the upward motion in the cloud be continued as long as air highly charged with vapor runs in below.

It is also easy to explain why the base of the cloud, during this rapid motion of the air upwards, remains nearly at the same level; for, so long as the dew-point of the air at the surface of the earth, which supplies the ascending column with vapor, remains the same if the temperature of the air is the same also, it will have to ascend to the same height, before cold enough is produced by expansion to cause condensation of the vapor. This height is found to be about 100 yards for every degree between the temperature of the air and the dew-point, unless when the cloud becomes of great perpendicular depth, when the base sinks a little from the levity of the cloud.

The quantity of depression of the vapor as well as of the air, after due allowance is made for the fall of the dew-point from the expansion, is nearly 400 yards for a depression of the barometer one inch, on the supposition that the temperature of the air sinks 4 deg. for a diminution of the pressure equal to an inch of mercury.

A depression of near two and a half inches has been recorded in a spout accompanied with hail nine inches deep; hence the base of the cloud would descend in the centre near 1,000 yards; and of course, if the dew-point and temperature of the air were not more than 10 deg. apart, the cloud would reach the earth, and exhibit itself in the form of an inverted cone, expanding as it goes up, and by its rapid motion carrying up large drops of rain above the region of perpetual congelation, and throwing them out at the sides, in the form of *hail.*

From the principles here developed, it will be easy, when a great storm springs up, or comes within disturbing influence, to tell in what direction it is raging; for the direction of the wind points it out. An exception to this general principle was mentioned and explained in our last report. The connexion also between volcanoes and rains is no longer mysterious: for an upward motion of the air is produced and kept up by the volcano, the result of which must be a condensation of vapor, unless the dew-point is very low. So powerful is this tendency, that in South America a dry season is sometimes changed into a rainy one by the bursting out of a volcano. From these brief hints, we trust it will be acknowledged that something has been gained explanatory of the phenomena in question. But we must not stop here: it is not enough to know *where* it is raining at a given time; we must know *when* it will rain where we are. For this purpose, it is of primary importance to know the direction in which storms move, and also their velocity in all the different seasons of the year.

We would suggest as the most effectual, and perhaps the only, means of obtaining this end, an appropriation by Government for the purchase of meteorological instruments, to be presented to those academies, schools, and colleges, that would pledge themselves to keep a journal of the weather, according to a prescribed plan, for five years, and send a monthly statement to a meteorologist to be appointed by the Government."

9. *The Epidemic Yellow Fever of Natchez. In Medio-veritas. An Essay read before the Jefferson College and Washington Lyceum, December 2d,* 1837. By J. W. MONETTE, M. D.

The first 50 pages of this essay are chiefly devoted to a general survey of the facts which relate to the remote causes of Yellow Fever. We say *causes*, in as much, as the author is of the school of Hosack, and believes the concurrence of an indigenous and a foreign cause to be necessary to the production of the disease. Thus the arrival of a vessel with a certain kind of atmosphere, at a place where heat and moisture and filth, and a crowded population, have already contaminated the air, may be followed by the developement of the fever, when, neither would of itself, have been sufficient. This imported atmosphere acts as a ferment or leaven. We have not time or space, at present, to inquire into the reality of this hypothesis; but that our readers may do it for themselves, we shall present our author's doctrine in his own words.

"*Corollaries.*—If the views herein advanced be correct, the following are legitimate deductions.

I. That the miasm, or gaseous matter which is essential to the production of epidemic yellow fever, is generated only while the extreme temperature in the shade, is at least up to 88 deg. of Fahrenheit; and that so long as there is sufficient agitation and change of the air by winds, it will not accumulate in sufficient quantity to produce yellow fever as an epidemic: that when sufficient miasm is produced and accumulated,the malarious combination, which likewise requires several days of calm, sultry weather, will proceed at a still lower temperature.

II. That the miasm of yellow fever, is, *per se*, in a pure atmosphere, probably innoxious; but acquires active morbific properties by combining with sultry, hot air, which has been exhausted by respiration, and charged with human exhalations; and that it then becomes *malaria*, or infectious air, which is an active predisposing cause, as well as a proper "*nidus*" for the reception and evolution of the infection of yellow fever.

III. That this malarious condition of the local atmosphere of any city, or portion of a city, may be so concentrated as to produce a strong predisposition to yellow fever in many of the inhabitants, without actual disease, until a few cases are excited into action by highly exciting causes, when *infection* is generated, and speedily the malarious district becomes the infected district: which result would have been prevented by a storm, or change of weather previous to those cases.

IV. That when the malarious combination is sufficiently concentrated for this purpose, a large quantity of *infected air*, brought from an infected district, or a large number of cases of yellow fever introduced from another point, will convert that malaria into infected air, and produce an epidemic likewise.

V. That, consequently, although yellow fever is very often a disease of local origin, it *may*, under peculiar circumstances, be carried from one city to another, and there propagated.

VI. That accordingly epidemic yellow fever may be averted sometimes by one or all of the following measures, enforced at a time, when, according to the principles herein set forth, the malaria is forming rapidly; viz:

1. By a dispersion of the greater portion of the citizens to the country.

2. By removing from the city, and especially from the districts usually infected, all *strangers*, or those who have not become acclimated by a residence of two or three years, and who would in course be the first attacked.

3. By prohibiting the introduction from foreign places of infected air, or *fomites*, or patients laboring under yellow fever, during the prevalence of malarious accumulations.

VII. That the vicinity of any of the large bayoux, or gullies about Natchez, is more dangerous as a residence than more remote points: that these ravines are the reservoirs in which the malaria mostly accumulates before it is dispersed through the city by gentle winds: of course persons should avoid them in the sultry, autumnal months.

VIII. That this miasm, or even the malaria, is not the result of animal or vegetable decomposition; or of exhalations from city filth and such like sources.

IX. That the general atmosphere in the country may be as healthy as ordinary, while in the city yellow fever may be sweeping off the population like a pestilence; of course, that the disease is the result of some local atmospheric contamination.

X. That when the infection has spread, or is beginning to spread, the only safety for those who are strangers, or unacclimated to yellow fever infection, is speedy flight: for no disinfecting agents heretofore known, or tried, possess any power to destroy the infection of yellow fever in the general air, cold alone excepted.

XI. That those who seek safety in flight, should carry with them as few bulky, light articles, of a woollen or porous texture as practicable, lest they might generate an infected air in their retreat. That they should not return to their houses until after cold winds and frosts, during which their rooms, bedding, &c., have been freely exposed to ventilation and to the action of the frost."

The most valuable part of the essay of Dr. Monette is the narrative, or that which gives a "sketch of the history of the different yellow fever epidemics of Natchez," from 1817 to 1837, a period of 20 years. Throughout this lengthened term, the years in which the disease prevailed as an epidemic, were 1817, 1819, 1823, 1825, 1829 and 1837. With much industry and apparent fidelity, Dr. Monette has collected a large mass of meteorological and other observations, which seem to have a bearing on the production of the fever; and make an important contribution to the medical history of Natchez and its vicinity. We could wish that the other towns of the great Valley had historians of equal zeal and ability with our Mirsissippi friend.

